# *In silico* and *in vitro* approaches allow the identification of the Prosystemin molecular network

**DOI:** 10.1016/j.csbj.2022.12.006

**Published:** 2022-12-07

**Authors:** Roberto Natale, Mariangela Coppola, Nunzio D'Agostino, Youjun Zhang, Alisdair Robert Fernie, Valeria Castaldi, Rosa Rao

**Affiliations:** a Department of Agricultural Sciences, University of Naples Federico II, Portici 80055, Italy; b Center of Plant Systems Biology and Biotechnology, Plovdiv 4000, Bulgaria; c Max-Planck-Institut für Molekulare Pflanzenphysiologie, Potsdam-Golm 14476, Germany; d Interuniversity Center for Studies on Bioinspired Agro-Environmental Technology (BAT Center), University of Naples Federico II, Portici 80055, Italy

**Keywords:** Protein-protein interaction, Prosystemin interactors

## Abstract

Tomato Prosystemin (ProSys), the precursor of Systemin, a small peptidic hormone, is produced at very low concentration in unchallenged plants, while its expression greatly increases in response to several different stressors triggering an array of defence responses. The molecular mechanisms that underpin such a wide array of defence barriers are not fully understood and are likely correlated with the intrinsically disordered (ID) structure of the protein. ID proteins interact with different protein partners forming complexes involved in the modulation of different biological mechanisms. Here we describe the ProSys-protein network that shed light on the molecular mechanisms underpinning ProSys associated defence responses. Three different approaches were used. *In silico* prediction resulted in 98 direct interactors, most clustering in phytohormone biosynthesis, transcription factors and signal transduction gene classes. The network shows the central role of ProSys during defence responses, that reflects its role as central hub. *In vitro* ProSys interactors, identified by Affinity Purification-Mass Spectrometry (AP-MS), revealed over three hundred protein partners, while Bimolecular Fluorescent Complementation (BiFC) experiments validated *in vivo* some interactors predicted *in silico* and *in vitro*. Our results demonstrate that ProSys interacts with several proteins and reveal new key molecular events in the ProSys-dependent defence response of tomato plant.

## Graphical abstract

**Figure:**
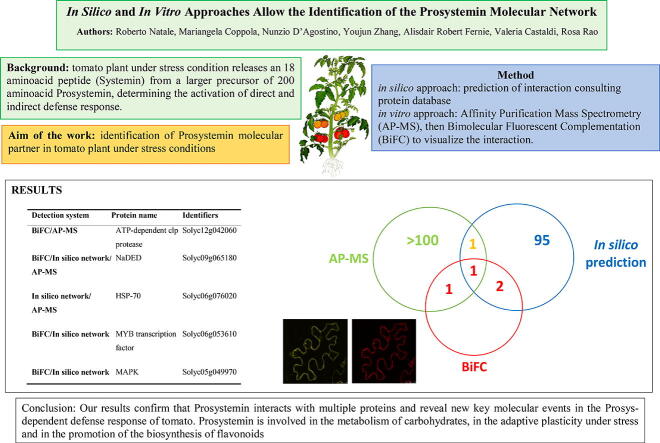

## 1 Introduction

Proteins are the basis of most of the biological processes that determine the functioning of living organisms where they generally act in stable or transient complexes. Thus, large-scale identification of protein–protein interactions (PPIs) provides crucial insights into how biological pathways are structured and coordinated by individual protein functions. PPI networks give essential information on the regulation of plant developmental processes as well as plant responses to environmental stimuli.

The responses to environmental stresses require adaptations at cellular and molecular levels that involve kinase cascades, reactive oxygen species and phytohormones. Such complex signalling networks are based on the cooperation of thousands of molecules, including proteins, nucleic acids, lipids, and others [1], [2].

PPIs studies have revealed complex networks covering all scientific fields from medicine and pharmacology, such as the mechanism of insulin action [3], to the complex metabolic processes that take place in both animal and plant cells [4], [5].

The coverage of PPIs is presently improved by a variety of computational methods [6 and reference therein] also thanks to the development of *in vitro* and *in vivo* techniques, capable of detecting previously uncharacterized or novel PPIs.

*In vitro* techniques are mainly based on cloning and recombinant expression of proteins and on the identification of physical contact between proteins or between proteins and specific antibodies [7]. These techniques include affinity purification mass spectrometry (AP-MS), yeast two-hybrid (Y2H) and Co-immunoprecipitation (Co-Ip).

The putative interactors detected through these approaches must be validated using *in vivo* techniques, which allow real time monitoring of interactions, as the host organism, in which the PPI takes place, is alive when the analysis is carried out. Because of its specificity and immediacy, bimolecular fluorescent complementation (BiFC) is the most widely used technique for direct visualization of PPIs in living cells. Indeed, the BiFC assay is based on the reconstitution of an intact fluorescent protein when two proteins are brought together due to their interactions [8]. Conversely, *in silico* prediction of PPIs is achieved through computational methods capable of determining reliable potential interactions to be further verified using experimental approaches. Numerous *in silico* methods have been developed to predict new interactions or to confirm already identified interactions [9]. In addition to other databases, PPI studies on tomato (*Solanum lycopersicum, L.*) can make use of the Predicted Tomato Interactome Resource (PTIR) and of the Search Tool for the Retrieval of Interacting Genes/Proteins (STRING). Both resources were used in this study.

PTIR is based on experimentally determined orthologous interactions in six model organisms. It covers more than 300,000 non-redundant PPIs with different confidence levels [10]. More than 30 % of the tomato protein complement is represented in the database. STRING is one of the most comprehensive databases, as it includes information on direct (physical) and indirect (functional) protein associations for over 2000 organisms. PPIs in STRING derive from genomic context predictions, high-throughput experiments, interactions aggregated from other (primary) databases [11]. As for *S. lycopersicum*, the current version (v. 11.5) covers 4,908,531 pairwise interactions.

Additional software allows the analysis of large amounts of protein–protein and protein-nucleic acid data and the visualization of biological networks in a simple way such as graphs, in which the nodes represent biomolecules and the edges the relationships between them. Among these, Cytoscape is an open-source tool for visualizing interaction networks [12], integrating these with attribute data (e.g., expression profiles, phenotypes, etc.), and linking them to functional annotation resources [13].

In tomato plants, after wounding or insect attacks, the octadecapeptide systemin (Sys) is released from its precursor, ProSystemin (ProSys), to trigger defence responses via the octadecanoid pathway [14], [15]. Sys was the first plant hormone peptide discovered in 1991 by Ryan’s team, following a pioneering study which demonstrated that the peptide is a potent inducer of proteinase inhibitors (PIs) in tomato and potato plants. Several studies have shown that Sys endows tomato plants with resistance against multiple biotic and abiotic stressors. For example, constitutive expression of the *ProSys* gene in tomato plants has been observed to trigger the increase in PIs and other defensive compounds that confer resistance to chewing and sucking insects, phytopathogenic fungi and salt stress [14], [16], [17], [18], [19], [20], [21]. In addition, transgenic plants have been characterized by an increasing level of indirect defence barriers with a consequent greater attractiveness towards natural enemies of phytophagous insects [22], [23], [24]. However, the molecular basis of such a great 'anti-stress' ability has not been fully explained. It has recently been shown that ProSys is an intrinsically disordered (ID) protein [25], a class of proteins with the ability to interact with different molecular partners in many-to-one and one-to-many binding equilibria (i.e., acting as “hub”) [26].

The interaction of ProSys with diverse partners might modulate numerous outputs, thus explaining the multiple resistances observed in transgenic plants. Sys is known to interact with 3 different partners: the Sys receptors SYR1 and SYR2 [27], and SR160 [28], a member of the leucine-rich repeat (LRR) receptor kinase family, very similar to the brassinolide receptor kinase BRI1, previously referred to as Sys receptor [29], [30]. However, as far as we know, there are no known ProSys interactors. Therefore, in this work we tried to identify ProSys protein partners, using both *in silico* and *in vitro* approaches and validating some interactors by *in vivo* studies. The main findings indicate that the strategy adopted was successful, allowing us to describe the ProSys PPI network for the first time.

## 2 Results

### 2.1 Description of the ProSys sub-network and *in silico* identification of its main interactors

In a previous work [16], just over 500 differentially expressed genes (DEGs) were identified by comparing wild-type and transgenic tomato plants constitutively expressing *ProSys* (RSYS). These DEGs were used to query PTIR and STRING with the aim of identifying putative interactors. In addition, for each DEG the corresponding ortholog was found in *Arabidopsis thaliana*, in order to take advantage of the wealth of information associated with the protein complement of this model plant. A list of 309 *A. thaliana* proteins was obtained from the over 500 tomato DEGs.

All captured interactions were combined and assessed based on the sharing of gene ontology terms, on co-expression, co-localization, and availability of interacting protein domains. Furthermore, for each interaction the level of confidence was assessed by evaluating the evidence supporting direct contacts [31], [10]. A non-directional network was constructed for each dataset obtained by querying the two databases. The STRING and PTIR datasets resulted into a network of 15,642 and 3,334 nodes, respectively. This substantial difference depends on the different sizes of the queried databases.

Then, the individual networks were collapsed into a single and complex network consisting of 16,002 nodes (proteins) and 163,627 edges (PPIs) (supplementary materials, Fig. S1). Finally, a sub-net was extracted by selecting the node corresponding to the ProSys protein (Solyc05g051750) and all direct interactors. The topology of the network was then investigated by evaluating the following parameters: the degree distribution (Fig. 3a) that is the probable distribution of the number of edges that are incident at each node of the network; the betweenness centrality (Fig. 3b) which measures the amount of influence a node has on the flow of information in a graph (i.e., the network): for a node, having a high centrality value, this implies that it is crossed by many paths and becomes an obligatory passage between many nodes; the clustering coefficient distribution (Fig. 3c) that is a measure of the degree to which nodes in a graph tend to cluster together. The values of network parameters are listed in Table C (supplementary materials). The degree distribution reveals a “scale-free” network characterized by the presence of large hubs (i.e., a few nodes strongly connected to other nodes in the network). Indeed, the network includes nodes with scores ranging from 1 to 4,366. This means that nodes with much higher degree distribution scores are essential for maintaining the structure of the network. For example, the highest score (4,366) was assigned to the node corresponding to the RNA polymerase enzyme (Solyc02g083350)(that is not a ProSys direct interactor but, likely a consequence of the plant’s need to reprogram the transcriptome in response to the constitutive expression of *ProSys* cDNA. The ProSys’s node, indicated in Fig. 3 with blue arrow, is crossed by many paths and this reflects its crucial role for the structure of the entire network. Other nodes as those corresponding to the heat shock protein Solyc06g076020 and the MYB transcription factor Solyc06g053610 are indicated in the Fig. 3 with orange and green arrows because they are crossed by many paths.

**Fig. 1:**
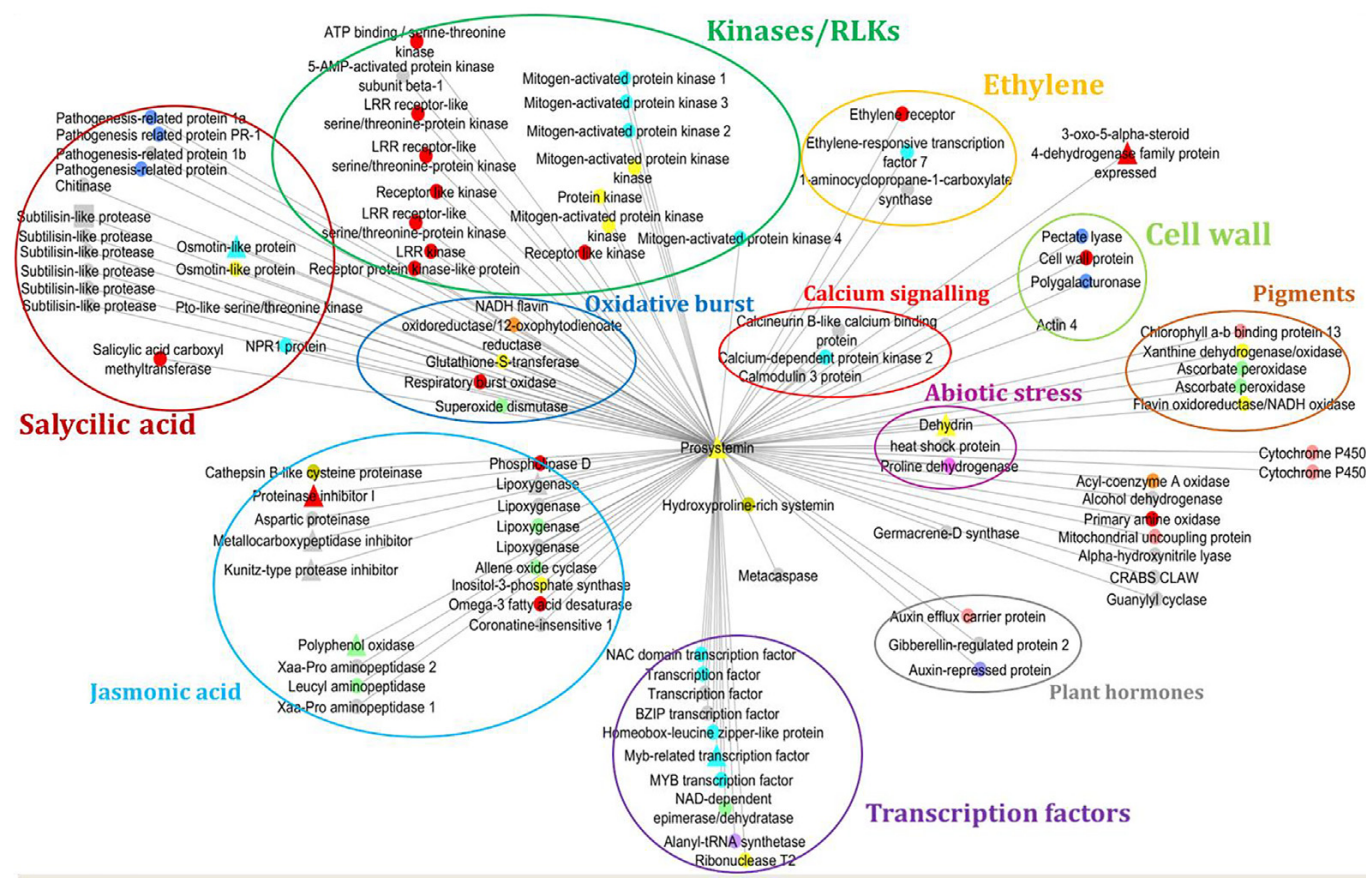
ProSys sub-network. The nodes were coloured based on the cellular localization of the protein. The up-regulated genes (N. = 10) are shown with triangular shape; the square shape is reserved for the down-regulated genes (N. = 1) by *ProSys* over-expression, and the circular shape (N. = 88) for putative interactors.

The resulting ProSys sub-network is shown in Fig. 1 where the different colours of the nodes indicate the cellular localization of the proteins. Eleven nodes represent proteins encoded by DEGs (triangles and squares) while the other putative interactors, indicated with circular shape, come from the queried interactomes.

The ProSys sub-network consists of 99 nodes and 98 interactions (Fig. 1). The size of the nodes is uniform because only the interactions in which ProSys is involved were shown.

For this reason, all the nodes have a degree equal to 1, except for ProSys for which it is 98. The 98 ProSys interactors (listed in Table A, supplementary materials) were classified according to their role in regulation and participation in defence mechanisms through a careful analysis of the literature. As a result, protein clusters with a specific role in defence were highlighted. The interactors were divided and classified in Gene Ontology (GO) categories (elliptic and circular coloured shape), highlighting several classes of defence-related proteins. Oxidative burst (light blue cluster) and calcium signalling (red cluster) are the first line of defence activated in plants under attack. These clusters included four and three interactors, respectively.

Other additional interactors were several MAPKs (fluorescent green) and proteins related to salycilic acid (SA) and jasmonic acid (JA) pathways (red and blue, respectively). SA is known to mediate host responses to pathogen infection [32]. The SA-related protein cluster included six subtilisin-like proteases, an osmotin-like protease, and the SA receptor NPR1 [33]. JA plays a crucial role in inducing systemic responses to herbivory [21], [34], [35], [36]. The JA-related protein cluster comprised four lipoxygenases and protease inhibitors. A further cluster of defence-related interactors (green) was associated with cell wall, whose reorganization generally follows the attack of a parasite, with the aim of preventing/reducing its penetration into plant tissues. Putative interactions of ProSys with the ethylene pathway (dark blue) have been also found; these include an ethylene receptor and an ethylene-responsive transcription factor, both of which are involved in the plant defence response.

Interestingly, the cluster of transcription factors (pink), included 10 proteins likely involved in the activation of defence-related genes. Other putative interactors were associated with abiotic stress (fuchsia), plant hormones (grey), and pigment metabolic pathways (orange). Fig. 1 also shows a list of coloured squares indicating the different sub-cellular localization of the proteins.

### 2.2 Experimental identification of ProSys interactors

The experimental identification of ProSys interactors was achieved by AP-MS screening the protein complexes formed *in vitro*. Both mCherry and ProSys-mCherry were expressed and purified from *Escherichia coli*. The recombinant proteins were incubated with total tomato proteins to perform the affinity interaction assay. Subsequently, all interactors were measured by Liquid chromatography–mass spectrometry (LC-MS) with high protein intensity of ProSys (Solyc05g051750). From the interpretation of mass spectra, more than three hundred proteins able to bind ProSys were identified. However, most of these proteins were either not characterized or their function was poorly documented. Given that large amounts of ribosomal proteins and translation-related proteins were detected by the AP-MS, these interactors were filtered out. Normalized signal intensities were processed to determine fold-change abundance (FC-A) scores.

Candidate interactors with a fold change greater than four, around seventy, were selected [37]. Information on functions, role in defence response and subcellular localization of these proteins were retrieved from UniProt, KEGG and Ensemble Plants and are listed in Table B (supplementary materials).

We next turned our attention to cytoplasmic proteins, as ProSys is a cytosolic protein [38]. Several cytoplasmic interactors showed high scores: the phosphogluconate dehydrogenase 2 (PGD2; Solyc05g010260), the syntaxin-like protein (Solyc12g089150), the heat shock protein (HSP; Solyc07g065840), two oxidoreductase enzymes (Solyc05g010260; Sloyc11g010960) and a calcium ion binding protein (Solyc01g099770). Intriguingly, one interactor is involved in the ethylene biosynthesis process (Solyc02g036350).

Four interactors previously identified *in silico* were also found by AP-MS. Two of them were characterized by high scores: NaDED (Solyc09g065180; FC-A = 36.64) and HSP (Solyc06g076020; FC-A = 10.28). Although the proteins interacting with unfolded peptide like heat shock proteins may be artifact of AP-MS [39], the presence of heat shock protein in the network predicted *in silico* encouraged us in considering this protein a good candidate. The remaining two interactors had FC-A scores below the fixed cut-off: the inositol-3-phosphate synthase (Solyc04g054740; FC-A = 1.38) and the alanine-tRNA ligase synthetase (Solyc01g111990; FC-A = 1.79).

### 2.3 *In vivo* validation of interactions

Four proteins, identified both *in silico* and *in vitro*, were selected for *in vivo* studies aiming to validate the interactions. In addition, a fifth protein was selected from the *in silico* network, a MAP kinase, due to the essential roles it plays in responses to biotic and abiotic stress. Another of them is NAD-dependent epimerase\dehydratase (Solyc09g065180) enzyme. This enzyme uses nucleotide sugar substrates for a variety of chemical reactions including those of the ROS scavenging system to alleviate oxidative damage [40]. In addition, the enzyme activity was associated with cell surface properties, exoenzyme production, and virulence of bacterial diseases [41].

We also selected two additional proteins associated with stress damage/response: a MYB transcription factor (Solyc06g053610) involved in the control of various processes, including plant responses to biotic and abiotic stress and defence [42], [43] and a MAPK-6 (Solyc05g049970) responsible for the regulation of the defence signalling cascade [44], [45]. MYB transcription factors represent one of the largest protein families in plants characterized by highly conserved N-terminal MYB DNA-binding domain repeats. MYB proteins have been particularly well studied as regulators of phenylpropanoids, that play key roles in plant defence both as components of chemical barriers and as defence signalling molecules. In addition, MYB proteins also contribute to salt tolerance in plants both regulating ABA synthesis and modulating cuticle formation and antioxidant defence [46], [47].

MAP kinases (MAPK) take part in one of the most studied mechanisms that plays a crucial role in eukaryotic systems linking perception of external stimuli with changes in cellular organization or gene expression: phosphorylation has a fundamental role in the progression of the signal through the MAP kinase cascade. A typical MAPK cascade consists of at least three serine/threonine kinases, which act in sequence. MAPKs are clearly involved in JA, SA and ET signalling pathways, the three major phytohormones with essential roles in both biotic and abiotic stress responses [48], [49], [50].

Finally, based on the score of AP-MS, we selected an ATP-dependent clp protease (Solyc12g042060), an enzyme often expressed as part of the cellular proteostasis, responsible for the breakdown of misfolded or damaged proteins within the cell during various stressful conditions [51].

**Fig. 2:**
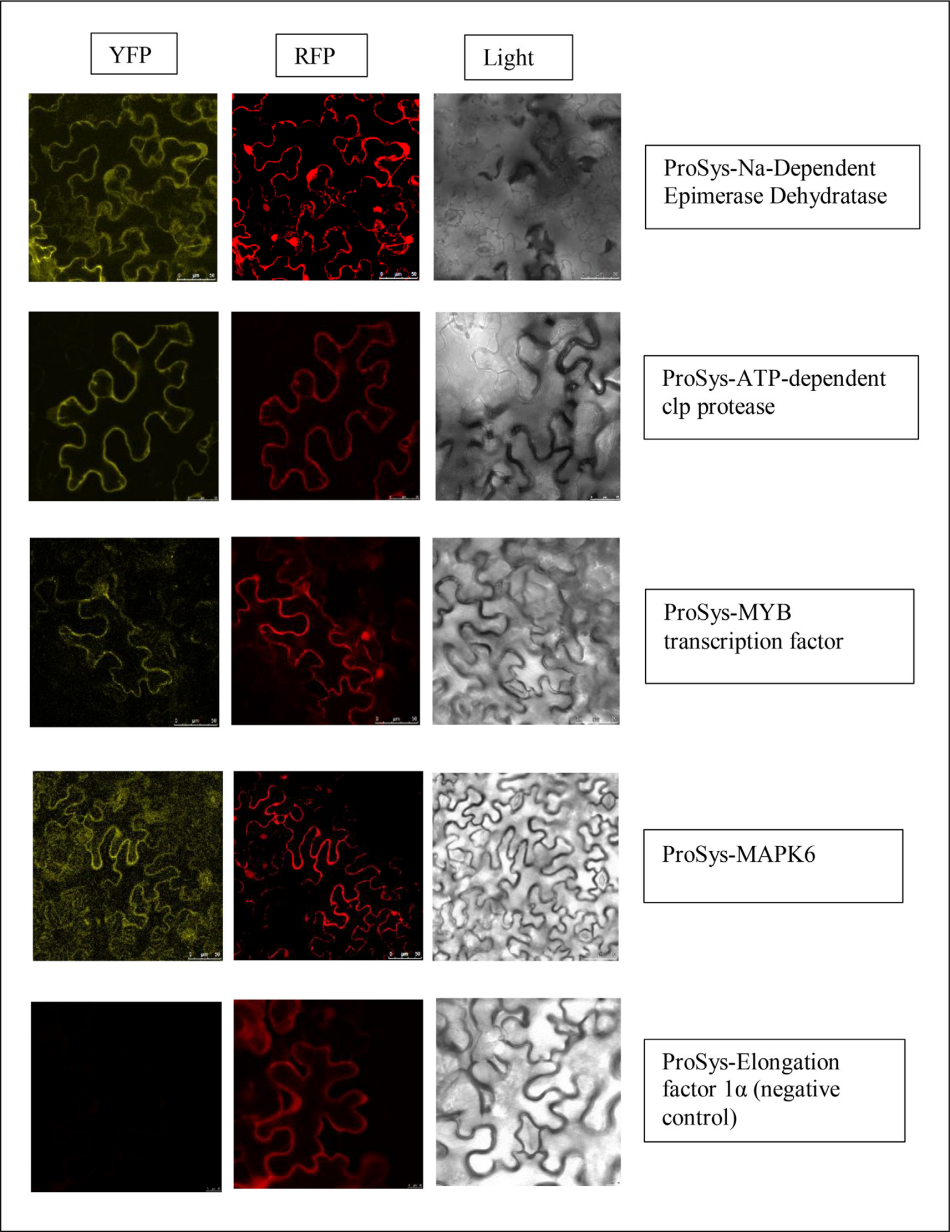
Confocal microscope images of ProSys interactions. The interaction between the proteins gives a yellow fluorescence signal due to the fusion of two non-fluorescent fragments of yellow fluorescence protein (YFP) (a). The reconstitution of YFP from its fragments (YFP-N, N-terminal fragment [amino acids 1–155]; YFP-C, C-terminal fragment [amino acids 156–239]) is the result of the interaction between the proteins. Furthermore, the vectors also contain a red fluorescent protein (RFP), used as control to verify the expression of the protein inside the cell; the exposure to different wavelengths determined different color emission: a) excitation of YFP (490–515 nm); b) excitation of RFP (555 nm); c) blank: same focal plane without laser excitation.

The results of the infiltration of young leaves of *N. benthamiana* with the recombinant vectors are reported in Fig. 2 and listed in Table 1. For all protein pairs tested, BiFC signals were detected in the cytoplasm and nucleus.

**Table 1:** List of ProSys interactors detected with different methods.

Detection system	Protein name	Identifiers
AP-MS/BiFC	ATP-dependent clp protease	Solyc12g042060
*In silico* network/AP-MS/BiFC	NaDED	Solyc09g065180
*In silico* network/AP-MS	HSP-70	Solyc06g076020
*In silico* network/BiFC	MYB transcription factor	Solyc06g053610
*In silico* network/BiFC	MAPK6	Solyc05g049970

## 3 Discussion

Protein-protein interactions mediate a wide range of biological processes, including the control of development and metabolism, and cell–cell interactions [9]. At the molecular level, PPIs are involved in post-transcriptional modifications, protein phosphorylation, recruitment of transcriptional co-factors, and are the main actors of many physiological and pathological processes [52], [53] such as signal transduction and defence responses [54], [39].

**Fig. 3:**
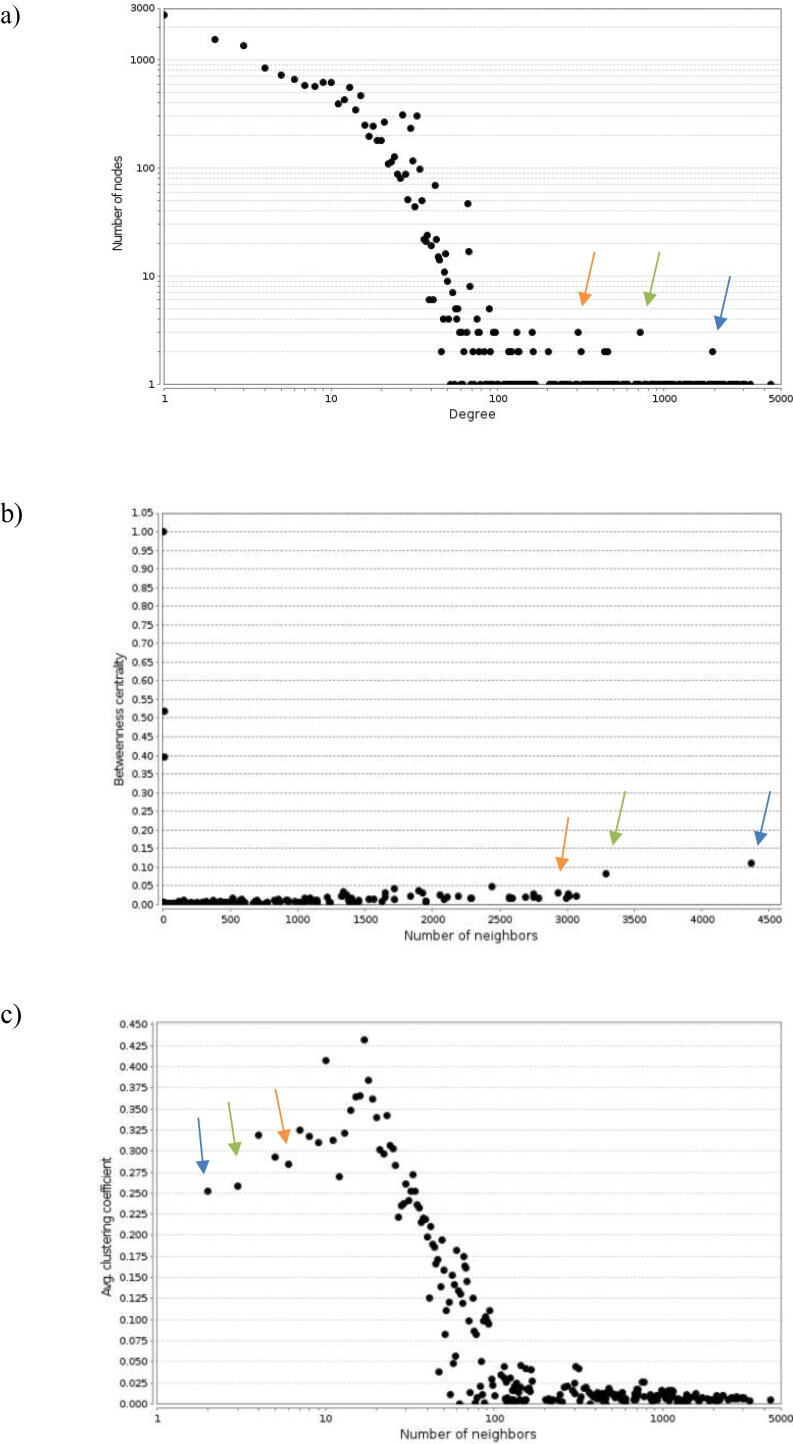
Network parameters. The image shows the network parameters analysis carried out in Cytoscape: a) Connection degree distribution; b) Betweenness centrality; c) Average Clustering Coefficient distribution. The blue arrows indicate ProSys, orange arrows indicate the Heat Shock Protein (Solyc06g076020) and the green arrows the MYB-related transcription factor (Solyc06g053610).

The study of protein interactions has undergone a great boost by exploiting omics data and when bioinformatics has become essential to study the biological functioning of PPI networks [55]. By using these tools, we developed the *in silico* ProSys network characterized by the presence of almost 100 ProSys direct interactors. The network shows the central role of ProSys during defence responses, confirmed also by the parameters used to describe the network (connection degree distribution, betweenness centrality, clustering coefficient distribution; Fig. 3) that reflects its role as central hub in the network. Similarly, the nodes corresponding to HSP and MYB transcription factor have a high degree of connection and can be considered as important hubs for the network.

Notably the ProSys network does not include SYR1 and SYR2, the two LRR-RKs that binds Sys [27]. These receptors likely have affinity only for the Sys peptide; in fact, the three-dimensional folding of ProSys, which exhibits a high index of disorder [25], can hide the Sys peptide located in the C-term region of the pro-hormone.

### 3.1 ProSys interacts with genes involved in early defence responses

ProSys-mediated defence system begins with the oxidative burst, one of the first defence reactions of plants with consequent rapid and transient production of a massive amount of reactive oxygen species, up to the modulation of phytohormone pathways and defence-related genes through the activation of transcription factors*.* Over-expression of ProSys produces a cytoplasmic change in calcium ion concentration, which activates calcium-sensitive proteins [20], [56]. This observation is in good agreement with the presence in the network of calcineurin B-like (CBL, Solyc03g083320), calmodulin (CaM, Solyc03g098050) and the calcium-dependent protein kinase 2 (CDPK2, Solyc04g009800), highlighted in red ovals in Fig. 1. CBL and CaM are small proteins containing multiple Ca^2+^ binding domains, which, by binding to Ca^2+^, transduce the signal to target proteins [56], [57], [58]. The key role of ProSys in early defence response is also supported by the large number of predicted interactors annotated as kinases (as MAPK6), receptor-like kinases, and LRR-receptors. Mitogen-activated protein kinases (MAPKs) are key components of plant defence pathways, playing critical roles in both basal defence and more specific interactions. These interactions could play a key role in signal transmission with the consequent activation of phytohormone biosynthetic pathways and the subsequent transcription of hormone-activated defence genes. For example, members of the Leucine-Rich Repeat (LRR) Ser/Thr receptor-like kinases (RLKs), generally located on the cell membrane, play an essential role in signalling during pathogen recognition mediated by Pathogen Associated Molecular Patterns, (PAMPs) and in subsequent activation of defence mechanisms [59]. These receptors determine the rapid activation of the MAPK signalling cascade and the production of ROS. ROS-related genes (blue cluster in Fig. 1) are induced as a result of the perception of plant stress. ROS are primary defence signals and are released a few seconds after damage is perceived. Superoxide anion (O^2–^), for example, is released locally in the damaged tissue, while hydrogen peroxide (H_2_O_2_) is produced both locally in the injured area and systemically throughout the plant [60].The NADPH oxidase enzyme (RBOH1, Solyc08g081690) contributes to the production of reactive oxygen species, a critical early signalling event connecting pattern-recognition receptors and intracellularly localized receptors-mediated immunity [61]. Glutathione-S-transferase (GST, Solyc01g099590) is a cytosolic enzyme that counteracts the damage caused by oxidative stress to the cell. It is an enzyme that catalyses the conjugation of toxic and hydrophobic chemicals to glutathione, increasing its solubility and promoting its sequestration in the vacuole or its transfer to the apoplast [62].

Several TFs are expected to interact with ProSys. Plant defence responses are triggered by TFs which regulate the transcription of genes involved in plant defence. For example, the MYB Solyc06g053610 is known to be involved in the response to biotic and abiotic stress [63]. MYB family members interact with transcription factors belonging to the WRKY family, implicated in plant defence and response to various environmental stresses [64], [65], [66], [67].

Notably, WRKY43, (Solyc12g042590) interacted with ProSys *in vitro*. WRKY TFs are involved in the regulation of various physiological programs in plants, including pathogen defence, senescence, trichome development and the biosynthesis of secondary metabolites [68].

The predicted ProSys-Hydroxyproline-rich systemin (HypSys; Solyc06g068520) interaction reinforces previous observations showing that they work cooperatively to produce a strong systemic response [69].

### 3.2 ProSys interacts with genes responsible for abiotic stress response

Previously it was observed that ProSys confers resistance to salt stress [19]. This observation correlates well with the predicted interaction with components of the plant responses to abiotic stressors (purple cluster in Fig. 1) such as dehydrin (Solyc02g084850), proline dehydrogenase (ProDH, Solyc02g089620), and heat shock protein 70 (HSP70, Solyc06g076020). Dehydrins are highly hydrophilic and thermostable IDPs, induced by various abiotic factors and phytohormones [70] they are up regulated in RSYS plants [16].

Their main function is to stabilize membranes, enzymes, and nucleotides in cells under abiotic stress [70]. The metabolism and accumulation of proline are associated with abiotic stress prevention mechanisms. For example, in *A. thaliana*, the proline level increases more than fifty folds in response to salt stress [71], [72]. The ProDH enzyme is involved in proline catabolism, which helps the plant regulate the proline level. Notably, ProDH is also a defence component against biotic stress, as it contributes to the hypersensitive response and disease resistance [73]. Heat shock protein 70 (HSP), a protein typically induced in response to high temperatures, has a chaperone function as improperly folded proteins accumulate under stressful conditions [74]. The interactors discussed so far support the previous data demonstrating that ProSys can improve tolerance against abiotic and biotic stress and enhances both direct and indirect plant defences [19], [22], [75].

### 3.3 Plant late defence response mediated by ProSys interactors

The Osmotin-like protein (Solyc08g080620) in the network is classified as PR-5 protein, which has been found to be up-regulated in RSYS plants [16]. Osmotin is a protein rich in cysteine residues and is involved in osmoregulation. It belongs to the PR protein family and has been used to produce transgenic plants resistant to fungi and tolerant to osmotic stress [76]. Xu and colleagues [77] demonstrated that not all PR proteins are involved in the same signalling cascades in tobacco; in fact, PR-5 was induced by both SA and ET/JA, while other proteins of the family are SA-specific [78]. The up-regulation of PR-5 compared to ProSys can be explained by the need to balance the different hormones and to counteract the attack of necrotrophic microorganisms, since the overexpression of osmotin causes cell death [76].

Since metacaspases are known to promote the induction of programmed cell death during biotic and abiotic stress [79], the ProSys-metacaspase interaction suggest a possible contribution of ProSys in programmed cell death following damage from stress [79]. Previous works have shown the involvement of ProSys in the modification of the Volatile Organic Compounds (VOCs) emitted by tomato plants with the consequent increase in the plant attractiveness of the natural enemies of herbivorous pests [80]. These observations are corroborated by the inclusion in the PPI network of the germacrene-D-synthase (Solyc12g006570.1), an enzyme involved in the biosynthesis of volatile terpenoids released by the plant following the attack of herbivores [81]. Corrado and co-workers [22], highlighted the contribution of *ProSys* in increasing the indirect defence level of plants by improving the attractiveness of parasitoids of herbivorous insects ascribed to the induction of germacrene-C-synthase, an enzyme involved in the production of sesquiterpenoids.

### 3.4 ProSys interactors associated with phytohormone biosynthesis

ProSys also appears to be related to the ET pathway. The ACS interactor (1-Aminocyclopropane-1-Carboxylate Synthase; Solyc01g095080) regulates the synthesis of ET, ensuring the formation of the 1-aminocyclopropan-1-carboxylic acid (ACC) precursor. The ERF7 (Ethylene-responsive transcription factor 7; Solyc06g065820) gene encodes the TF that controls the expression of ET-responsive genes following abiotic and biotic stimuli [82]. Five ERFs have been found to be overexpressed in *Solanum pimpinellifolium* under salt stress, and overexpression of ERF-7 increases salt tolerance [83]. The putative interaction of ProSys with the ACC enzyme, ERF elements and the ethylene receptor (Solyc09g075440) suggests that ProSys contributes to the crosstalk between the JA and ET pathways. As for the JA signalling pathway, several proteins (listed in Table A, supplementary materials) interact with ProSys, most of which are both early and late defence-related enzymes. The early and late wounding-responsive genes are regulated by distinct mechanisms. Local wounding leads to ProSys processing followed by binding of Sys to SYR1 and subsequent activation of genes involved in JA biosynthesis.

Four lipoxygenases (LOXs) were in the JA group. These enzymes are known to be involved in many plants developmental processes and in the regulation of stress responses. Indeed, the LOX pathway plays a key role in local and systemic wound response, releasing free radicals as early signals of defence and toxic metabolites as a preventive measure. In addition, LOXs modulate the plant response against abiotic stresses regulating plant hormones as SA, JA, ABA [84 and reference therein].

The increase in the level of endogenous JA leads to the activation of late JA-responsive genes that play a direct role in defence. Late defence genes include proteinase inhibitors (*PIs*), induced in wounded or insect-attacked leaves, which directly interfere with the assimilation of nutrients by the larvae*.* The JA group include polyphenol oxidase (PPO) an enzyme belonging to the jasmonate-dependent inducible defence system. Constabel and colleagues [85] demonstrated the induction of PPO activity in tomato leaves by the overexpression of *ProSys* and systemin, and the supply of methyl-jasmonate.

The presence of auxin and gibberellin regulatory enzymes as putative ProSys interactors opens new perspectives about the plastic role of these proteins in plant defence mechanisms. Auxin has long been recognized as a regulator of stress responses, with an interplay with other phytohormones. Indeed, it is well known that it interacts antagonistically with SA (biotrophic resistance) and synergistically with JA (necrotrophic resistance) [86]. Gibberellins are a class of growth hormones with a key role in response to environmental stimuli as light, temperature, salt, and biotic stress. The molecular mechanism is mediated by DELLA proteins under the regulation of gibberellin transduction pathways, reducing the levels of reactive oxygen species [87 and reference therein].

### 3.5 ProSys affinity with cell wall and pigment proteins

Plant cell wall is composed by a complex network of proteins trapped in a polysaccharide matrix that contributes to signalling events by interacting with both intra- and extra-cellular proteins [88]. Enzymes such as pectate lyases or polygalacturonases, found to be ProSys interactors, influence plant cell wall integrity during stressful events. Indeed, they contribute to pectin hydrolysis, generating molecules that act as plant immunity elicitors known as oligogalacturonides (OGs). Intriguingly, OGs-related pathway has been shown to be up-regulated in transgenic plants that overexpress the ProSys protein lacking the Sys sequence [89]. We can speculate that ProSys interaction with cell wall proteins can activate several cellular responses in local and distal tissues.

As regards the pigment group affinity with ProSys, is noteworthy the presence of ascorbate peroxidases (APXs), important components of the enzymatic antioxidant defence system used by plants to overcome the oxidative stress. Indeed, as suggested by Dietz in 2016 [90], APXs can interact with different molecular partners, such as proteins involved in the signal transmission after external stimuli. Further efforts are needed to clarify the role of plant APXs and how they interact to integrate stress signals.

Chlorophyll *a/b* binding protein 13 (CAB-13), an additional *in silico* detected ProSys interactor, belongs to the light harvesting complex of the photosystem II and appears to be involved in oxidative stress events and/or continuous light tolerance in tomato plants [91]. Intriguingly, its homolog in *A. thaliana,* named, LHCB3 is linked to the ABA signalling pathway under drought stress and stomatal closure [92]. This suggests that ProSys can act as a mediator also in salt and abiotic stresses [93].

### 3.6 *In vitro* and *in vivo* validation of ProSys interactions

The AP-MS experiments revealed a very large number of interactors confirming the complex scenario drawn by the *in silico* predicted network. Interestingly, the interaction of ProSys with NaDED was confirmed by the three different approaches used. NaDED is a member of a protein family with catalytic activity, involved in various biological processes such as rRNA processing, and positive regulation of translation and transcription. In addition, it is involved in the formation of carbohydrate derivatives by adding a carbohydrate residue to other molecules [94]. Sugars can stimulate plant immunity and promote the expression of defence genes [95]. For example, a high level of sugars in plant tissues improves the resistance of plants against pathogenic fungi [40]. This mechanism has been termed “high-sugar resistance”. It is important to note that sugars are the primary substrate that provides energy and structural material for defence responses in plants. Sugars trigger an oxidative burst early in the infection, inducing certain PR proteins. In addition, some sugars act as triggers by inducing greater resistance of plants to pathogens. Sugars can also act as intermediates, interacting with the hormone signalling network that regulates the plant immune system [40]. For example, several phytohormones including ET and JA, influence the sucrose signalling pathway [96]. For all these reasons, the ProSys-NaDED interaction could be associated with both sugar and plant defence signalling hormones. Among the possible interactors identified by AP-MS, several HSPs were found. Several interactors have catalytic activity, for example the enzymes encoded by Solyc05g010260 and Solyc11g010960 are oxidoreductases involved in redox processes during ET biosynthesis [97], [98], [99], [100] and under abiotic stresses such as floods, salinity, and drought. The crosstalk between JA, ET and SA signalling is thought to act as a mechanism for fine-tuning induced defence that is activated in response to multiple attackers. JA and ET interdependently and synergistically induce the expression of pathogen-responsive genes, such as defensins, to support plant tolerance against infections [101]. The latest ProSys interactor validated with BiFC was the ATP-dependent clp protease (Solyc12g042060). In the cytosol and nucleus of plant cells the damaged proteins are degraded by 26S proteasomes, whereas in chloroplasts and mitochondria this function is performed by proteases.

The main protease in chloroplasts is the ATP-dependent stromal Clp [102] which is overexpressed under (a)biotic stress and contributes to the digestion of misfolded proteins [103]. It is tempting to hypothesize that ProSys recruits the ATP-dependent clp protease which, thanks to its proteolytic activity, counteracts the toxic effects of damaged proteins.

SlMYB14 functions as a JA-responsive TF that plays a role in flavonoid accumulation and oxidative stress tolerance [104]. Flavonoids are secondary metabolites that could act as phytoalexins, compounds released by plants to ward off diseases and pathogens [105]. At the same time, ROS have additional signalling roles in plant adaptation to stress [106]. However, plants reduce ROS accumulation by altering the expression of ROS scavenging enzymes such as catalases, Cu-Zn-superoxide dismutases and peroxidases [107]. Therefore, the ProSys-MYB interaction could be responsible for activating the transcription of JA-responsive defence genes, reducing ROS accumulation, and promoting flavonoid biosynthesis.

## 4 Conclusion

Our results demonstrate that ProSys interacts with multiple proteins and reveal new key molecular events in the ProSys-dependent defence response of tomato such as the involvement in the metabolism of carbohydrates, in the adaptive plasticity of the plant under stress and in the promotion of the biosynthesis of flavonoids. Proteins are usually involved in interactions with an estimated average of 5–10 protein partners [108]. Our results suggest that ProSys is involved in a far greater number of interactions likely due to its ID structure. Understanding the functional role of the interacting complexes shown here will provide crucial insights into the ProSys-dependent defence mechanisms.

## 5 Materials and methods

### 5.1 *In silico* prediction of protein interactions

The 695 previously identified tomato DEGs [16] were used to query several plant PPI databases [10], [11]. BLASTx was performed (e-value 10^-5^) to search the DEGs against the *A. thaliana* RefSeq database. The resulting 309 *A. thaliana* proteins were used to predict PPI networks by querying the Search Tool for the Retrieval of Interacting Genes/Proteins (STRING; https://string-db.org). The list of tomato proteins was used to retrieve the PPI networks form the Predicted Tomato Interactome Resource (PTIR; https://bdg.hfut.edu.cn/ptir/index.html). The ProSys sub-network was selected and imported in Cytoscape 3.8.2 (https://www.cytoscape.org) to study the network topology. Each node of the network was described with several attributes (cellular localization, biological function and belonging to metabolic pathways) retrieved from the attribute files downloaded from the Ensembl Plant (https://plants.ensembl.org/index.html). After removing any duplicates and self-loops, the Cytoscape Network Analyzer Tool function has allowed the analysis of the graph through the automatic calculation of a series of parameters such as betweenness centrality; clustering coefficient; connections degree. The schematic representation of the procedure is represented in supplementary materials, Fig. S2.

### 5.2 CDNA cloning and vector construction

The *ProSys* gene was amplified from the pMZ vector [109] using specific primers with attB recombination sequence (Table 2), based on Gateway BP cloning system, and cloned in the pDONR221 Donor vector (Thermo Fisher Scientific, Waltham, MA). The gene-specific primers used did not include a stop codon to ensure C-terminal fusion of tags. Expression vector for AP–MS was constructed using the Gateway LR reaction with pET301 and pET300 vectors generating the expression cassette: pET301-mCherry-*Prosys*-HisTag and pET300-HisTag-*ProSys*-mCherry.

**Table 2:** List of primers used for *ProSys* cloning. The *Italic* style represent the attb adapter sequence.

Primer name	Sequence
ProsysFw	*AAAAAAGCAGGCTCCACC*atgggaactccttcatatgatatc
ProsysRv	*CAAGAAAGCTGGGTC*atagccgagtttattattgtctgtttgcat
attB1 adapter	5-GGGGACAAGTTTGTACAAAAAAGCAGGCT*ccacc*-3
attB2 adapter	5-GGGGACCACTTTGTACAAGAAAGCTGGGT*catagcc*-3
M13 Fw	GTAAAACGACGGCCAG
M13 Rv	CAGGAAACAGCTATGAC

The Destination vectors for BiFC-2in1 [110] were constructed using specific primers including sequences for the B1 and B4 regions, and B3 and B2 regions, respectively (Italic style in Table 3). The destination vectors used were pBiFCt-2in1-NN or pBiFCt-2in1-NC. *Agrobacterium tumefaciens* strain AGL1 was used for vectors expression.

**Table 3:** List of primers used for BiFC 2in1 cloning system.

Primer names	Sequence
Prosys Fw B1	GGGGACAAGTTTGTACAAAAAAGCAGGCTTAatgggaactccttcatatgatatc
Prosys Rv B4	GGGGACAACTTTGTATAGAAAAGTTGGGTGgagtttattattgtctgtttgcat
EF1α Fw B3	GGGGACAACTTTGTATAATAAAGTTGGAatgggtaaggaaaagattcac
EF 1α Rv B2	GGGGACCACTTTGTACAAGAAAGCTGGGTGcttccccttcttctgggcagc
NAD-dependent epim\dehyd Fw B3	GGGGACAACTTTGTATAATAAAGTTGGAatggctactcttgcttcttc
NAD-dependent epim\dehyd Rv B2	GGGGACCACTTTGTACAAGAAAGCTGGGTGgcactttcaggctttccaga
ATP-dependent clp protease Fw B3	GGGGACAACTTTGTATAATAAAGTTGGAatgcagtcaacaagcatcccatcg
ATP-dependent clp proteaseRv B2	GGGGACCACTTTGTACAAGAAAGCTGGGTGaaaatccaacttcccacaaaagca
MAP kinaseFwB3	GGGGACAACTTTGTATAATAAAGTTGGAatgaagaaaggatcttttgcacc
MAP kinaseRvB2	GGGGACCACTTTGTACAAGAAAGCTGGGTGtagctcagtaagtgttgccaatgg
MYB-related protein Fw B3	GGGGACAACTTTGTATAATAAAGTTGGAatgggtagagctccttgttg
MYB-related protein Rv B2	**GGGGACCACTTTGTACAAGAAAGCTGGGTGaaattctggtaattctggca**

### 5.3 Protein extraction for AP-MS

ProSys proteins were extracted from cells by Ultra-sonication and using a lysis buffer containing 20 mM of Phosphate-Buffered Saline (PBS) at pH 7.4, 20 mM NaCl, 5 % glycerol and 20 mM Imidazole and Phenylmethylsulfonyl fluoride (PMSF) 0.1 M. Three-weeks-old tomato plants (cultivar Moneymaker) were wounded on the upper side of the leaves, to simulate stressful condition, and harvested after 9 hours. Then, total proteins were extracted from wounded leaves using an extraction buffer, consisting of Tris HCl pH 7.5, 25 mM, MgCl2 15 mM, EGTA 5 mM, DTT 1 mM, PMSF 1 mM, NaCl 150 mM.

### 5.4 Sample preparation for AP-MS

The detailed materials and procedure were published by Zhang and co-workers in 2019 [37]. The total protein extracted from the leaves and the mCherry-ProSys complex were mixed in a 1.5 ml tubes with GFP-Trap® and gently mixed at 4 °C for 1 hour, to allow the formation of protein complexes.

Tubes were centrifuged to precipitate the beads coupled with the protein complexes and the supernatant was removed. The beads were recovered and washed with a specific buffer. The samples were ready for on-beads enzymatic digestion. The samples were dissolved in a small volume of 6 M urea/2 M thiourea pH 8, then 1 µl trypsin/LysC (0.4 μg/μl) was added. C18 Stage-SepPak® columns were used for desalination and peptide concentration, along with the Visiprep™ 12-Port Vacuum Manifolds and the vacuum pump. The peptides were dried in a SpeedVac™ evaporator and then they were resuspended in a final volume of 40 μl of resuspension solution (0.2 % TFA/5% acetonitrile) and transferred to a microtiter plate for mass spectrometric analysis. For this step, A Nano LC 1000 liquid chromatograph with a reversed-phase C18 column (Acclaim PepMap RSLC, 75 μm × 150 mm, C18, 2 μm, 100 A°) was used for this step.

### 5.5 LC-MS data analysis

LC-MS/MS analysis was performed on a Q Exactive Plus instrument (Thermo Fisher Scientific). Quantitative analysis of MS/MS measurements was performed with Progenesis QI software (Nonlinear Dynamics, Newcastle, UK). The proteins were identified from the spectra using Mascot (Matrix Science, London, UK) with the following parameters: TAIR10 protein annotation, requirement for tryptic ends, one missed cleavage allowed; fixed modification: carbamidomethylation (cysteine); variable modification: oxidation (methionine), peptide mass tolerance = ±10 p.p.m., MS/MS tolerance = ±0.6 Da, allowed peptide charges + 2 and + 3. A decoy database search was used to limit false discovery rates to 1 % at the protein level. Identifications of peptides below rank one or with a Mascot ion score less than 25 were filtered out. Mascot results were imported into Progenesis QI, the quantitative information about the peak area was extracted, and the results were exported for data plotting and statistical analysis.

For each protein, the corresponding Solyc identifier was retrieved by querying the UniProt database (https://www.uniprot.org). All entries were associated to GO terms (https://www.geneontology.org) and were assigned to KEGG pathways (https://www.genome.jp).

Ribosomal proteins and the translation-related proteins were filtered out at this stage. Normalized signal intensities were processed to determine the fold-change abundance (FC-A) scores, using the SAINT algorithm embedded in the CRAPome software [111], [112], [113]. Compared with the GFP control, background proteins were eliminated in the case of FC-A values of at least four within at least three replicates [114]. Compared with intensity of bait, only the proteins for which the intensity score was greater than 2 % should be considered as positive interactors.

Statistical analysis for this dataset was performed using the Student’s T-test.

### 5.6 BiFC analysis

Young leaves of four-weeks-old N. benthamiana plants were infiltrated with A. tumefaciens, transformed with BiFC vectors grown on selective medium, and protein expression visualized with DM6000B/SP5 confocal laser scanning microscope (Leica Microsystems, Wetzlar, Germany). Laser excitation was 490–515 nm for YFP and 555 nm for RFP; emission fluorescence was captured by 500 to 520 nm and 555 to 580 nm band-pass emission filters, respectively.

## 6 Authors statement

R.R. and M.C. designed the study, planned the experiments and supervised the work.

R.N. carried out the experiment with the supervision of R.R., M.C., Y.Z. and A.R.F.

R.R., N.D. and R.N. wrote the manuscript with the support of Y.Z., A.R.F. and V.C.

## Declaration of Competing Interest

The authors declare that they have no known competing financial interests or personal relationships that could have appeared to influence the work reported in this paper.
